# Pulpotomy for the Management of Irreversible Pulpitis in Mature Teeth (PIP): a feasibility study

**DOI:** 10.1186/s40814-022-01029-9

**Published:** 2022-04-02

**Authors:** Jan E. Clarkson, Craig R. Ramsay, Francesco Mannocci, Fadi Jarad, Sondos Albadri, David Ricketts, Carol Tait, Avijit Banerjee, Chris Deery, Dwayne Boyers, Zoe Marshman, Beatriz Goulao, Alice R. Hamilton, Katie Banister, Rosanne Bell, Lori Brown, David I. Conway, Pina Donaldson, Anne Duncan, Katharine Dunn, Patrick Fee, Mark Forrest, Anne-Marie Glenny, Jill Gouick, Ekta Gupta, Elisabet Jacobsen, Jennifer Kettle, Graeme MacLennan, Lorna Macpherson, Tina McGuff, Fiona Mitchell, Marjon van der Pol, Rebecca Moazzez, Douglas Roberston, Gabriella Wojewodka, Linda Young, Thomas Lamont

**Affiliations:** 1 Dental Health Services Research Unit, Dundee Dental School, The University of Dundee, 9th Floor, Park Place, Dundee, DD1 4HN UK; 2 NHS Education for Scotland, Edinburgh, UK; 3 Health Services Research Unit, University of Aberdeen, Aberdeen, UK; 4 Centre for clinical and translational sciences, King’s College London, London, UK; 5 School of Dentistry, University of Liverpool, Liverpool, UK; 6 Dundee Dental School, The University of Dundee, Dundee, UK; 7 Faculty of Dentistry, Oral & Craniofacial Services, Kings College London, London, UK; 8 School of Clinical Dentistry, University of Sheffield, Sheffield, UK; 9 Health Economics Research Unit, University of Aberdeen, Aberdeen, UK; 10 Patient and Public Involvement Health Services Research Unit, University of Aberdeen, Aberdeen, UK; 11 School of Medicine, Dentistry and Nursing, University of Glasgow, Glasgow, UK; 12 Edinburgh Dental Institute, NHS Lothian, Edinburgh, UK; 13 Division of Dentistry, University of Manchester, Manchester, UK; 14 Oral Clinical Research Unit, King’s College London, London, UK

**Keywords:** Irreversible pulpitis, Full pulpotomy, Root canal treatment, Primary care, Feasibility, Vital pulp therapy, General dental practice, PPI, Training

## Abstract

**Background:**

Progression of dental caries can result in irreversible pulpal damage. Partial irreversible pulpitis is the initial stage of this damage, confined to the coronal pulp whilst the radicular pulp shows little or no sign of infection. Preserving the pulp with sustained vitality and developing minimally invasive biologically based therapies are key themes within contemporary clinical practice. However, *root canal treatment* involving complete removal of the pulp is often the only option (other than extraction) given to patients with irreversible pulpitis, with substantial NHS and patient incurred costs. The European Society of Endodontology’s (ESE 2019) recent consensus statement recommends *full pulpotomy*, where the inflamed coronal pulp is removed with the goal of keeping the radicular pulp vital, as a more minimally invasive technique, potentially avoiding complex root canal treatment. Although this technique may be provided in secondary care, it has not been routinely implemented or evaluated in UK General Dental Practice.

**Method:**

This feasibility study aims to identify and assess in a primary care setting the training needs of general dental practitioners and clinical fidelity of the full pulpotomy intervention, estimate likely eligible patient pool and develop recruitment materials ahead of the main randomised controlled trial comparing the clinical and cost-effectiveness of full pulpotomy compared to root canal treatment in pre/molar teeth of adults 16 years and older showing signs indicative of irreversible pulpitis. The feasibility study will recruit and train 10 primary care dentists in the full pulpotomy technique. Dentists will recruit and provide full pulpotomy to 40 participants (four per practice) with indications of partial irreversible pulpitis.

**Discussion:**

The Pulpotomy for the Management of Irreversible Pulpitis in Mature Teeth (PIP) study will address the lack of high-quality evidence in the treatment of irreversible pulpitis, to aid dental practitioners, patients and policymakers in their decision-making. The PIP feasibility study will inform the main study on the practicality of providing both training and provision of the full pulpotomy technique in general dental practice.

**Trial registration:**

ISRCTN Registry, ISRCTN17973604. Registered on 28 January 2021.

Protocol version

Protocol version: 1; date: 03.02.2021

**Supplementary Information:**

The online version contains supplementary material available at 10.1186/s40814-022-01029-9.

## Background

The economic burden of dental disease to the NHS, patients and society is substantial, accounting for a global expenditure of $544.41bn annually [1]. NHS expenditure on dental care in England exceeds £3bn per year and £527 million in Scotland [2]. Most of this expenditure is due to dental caries, one of the most prevalent non-communicable diseases worldwide [3–6]. The consequences are cumulative [7, 8] and can negatively impact the quality of life and productivity.

Most adults experience decay, and the National Dental Epidemiology Programme for England 2018 reported that 90% of adults have at least one restoration [9]. Dental caries results in localised and progressive demineralisation of the dental hard tissue; if undisturbed, the bacterial insult will cause the pulp of the tooth to become inflamed and persistent inflammation can lead to *irreversible pulpitis* (when the vital inflamed pulp is incapable of healing) [10], *pulp necrosis* and *abscess* formation. Management of dental caries centres around primary prevention and/or operative intervention involving caries removal prior to the irreversible pulpitis stage. Preserving the pulp in a healthy state with sustained vitality, preventing apical periodontitis or abscess formation and developing minimally invasive biologically based therapies are key themes within contemporary clinical practice.

The recent position statement from the European Society of Endodontology (ESE 2019) [11] explains the challenge of managing deep caries and pulp exposure. If primary prevention and/or operative intervention involving caries removal prior to irreversible pulpitis fails, *root canal treatment* (*RCTx*) involving complete removal of the pulp is the only option (other than extraction) for patients with irreversible pulpitis, but it is a technically demanding procedure, especially in premolar and molar teeth and increases patient anxiety [12]. It is also time-consuming and costly to the NHS and patients. In Scotland, 111,000 RCTxs were provided in 2017/2018, costing £8.9m, and approximately 80% of this cost relates to RCTxs on premolar and molar teeth. Extrapolating these figures to England suggests the total cost of this treatment may be in excess of £71m per year.

Partial irreversible pulpitis is the initial stage of irreversible pulp damage, confined to the coronal (crown) pulp whilst the radicular (root) pulp remains vital, i.e. a healthy blood supply is maintained to the pulp tissue in the roots of the tooth. The full pulpotomy (FP) technique of removing the coronal pulp may keep the radicular pulp vital. Systematic reviews and randomised controlled trials suggest that pulpotomy may offer comparable treatment success rates and might be a cost-effective alternative to RCTx, but the evidence base is weak and sparse for the management of vital mature teeth with clinical signs of irreversible pulpitis in the UK NHS [13–16]. Two randomised controlled trials conducted in an adult population reported a success rate of pulpotomy comparable to RCTx (97.6% vs 98.3% at 1 year [17]; 85% vs 87.5% at 18 months [18]). However, both trials were conducted outside of the UK and in the secondary care setting. These studies have limited generalisability due to the lack of methodological rigour, difference in the health systems and the clinical approaches used. There is one ongoing trial in the UK [19] looking at this research question in a secondary care setting.

RCTx success rates vary considerably in the literature. A systematic review [20] together with recent studies conducted internationally [21–23], including primary and secondary care, concluded that the 2- to 10-year survival outcomes of RCTx ranged from 72 to 94.4%. Treatment success rates in primary care dentistry in Sweden, according to periapical status, was 62% immediately after treatment [24]; however, a review on RCTx survival in general dental services in England and Wales estimated 74% of root canal treated teeth pass through 10 years without re-treatment, apical surgery or extraction, and the success rate is above 90% in the first year [22]. Evidence from a systematic review and a retrospective follow-up study suggests that the success rate of pulpotomy for permanent posterior teeth may be over 90% at 1-year follow-up, but the participants in the included studies were not representative of UK NHS practice [13, 25].

The recently published commissioning standard in NHS England Restorative Dentistry defined the complexity of clinical and technical procedures according to levels of care 1, 2 and 3 (with increasing complexity). They also reflected the competency of clinicians and the equipment required to deliver care of that level of complexity [26]. Complex RCTxs are considered levels 2 and 3 and should be referred to specialists or dentists with special interest. FP could make the management of complex cases possible in primary care by general dental practitioners (GDPs), avoiding the need for extraction or the increased cost and burden on patients in referral.

FP is a novel technology for NHS primary dental care, and the Pulpotomy for the Management of Irreversible Pulpitis in Mature Teeth (PIP) feasibility study design has benefited from considerable active and informative input from patients in general dental practice, the Health Services Research Unit (HSRU) public involvement group and a national survey of GDPs and practitioners with research experience.

The importance of this topic to GDPs in the UK is clear from the responses to a survey hosted on the Scottish Dental Practice Based Research Network (SDPBRN) website, indicating that GDPs were very interested in the health technology to be tested in PIP but that clinical training would be required for both FP and RCTx. The survey showed that pulpotomy was not offered to NHS adult patients by 91% of the responding dentists with many citing contract restrictions and the costs of bio-ceramic materials as barriers. Overcoming these issues for PIP had already been discussed with UK Chief Dental Officers. Whilst the majority of dentists (97%) offered RCTx for uncomplicated teeth, this reduced to 68% and 20% for teeth with a moderate or complex risk of adverse outcomes, respectively. In addition, the PIP study team engaged with patient representatives and the public who highlighted the need to develop more modern dental techniques which aim to preserve rather than remove tooth tissue.

The COVID-19 pandemic has had a significant impact on the provision of dental care.

RCTx is commonly completed over several visits, whereas FP treatment is usually completed in one. FP may therefore reduce patient contact within a dental practice, as well as reducing the volume and cost of personal protective equipment required.

The PIP feasibility study has been designed to determine progression to a pragmatic, primary dental care, multi-centre, two-arm patient randomised control trial with an internal pilot comparing the clinical and cost-effectiveness of FP compared to RCTx in pre/molar teeth of adults 16 years and older showing signs indicative of irreversible pulpitis.

The following are the feasibility study objectives:

- To identify training needs of GDPs to undertake FP
- To develop a clinical training package for study GDPs
- To assess if the intervention can be optimally delivered in routine NHS practice
- To estimate the number of eligible patients per practice for the main trial
- To develop recruitment materials for the main trial accounting for patient and dentist views

## Methods/design

The PIP feasibility study is a multi-centre non-randomised intervention designed to determine if it is feasible to carry out FP in the UK NHS primary care setting.

### Study recruitment and allocation

#### Recruitment of dentists

We aim to recruit 10 GDPs with research experience from across the UK via our partner research networks and dentists who may or may not be active in other dental trials. Working with research-ready and experienced dentists will help us speedily identify training needs, service requirements and criteria for successful intervention delivery. A list of participating practices will be kept up to date and provided on the public trial website: https://w3.abdn.ac.uk/hsru/pip.

Following the expression of interest, an appraisal of each practice’s ability to recruit participants will be conducted including evidence of a sufficient supply of eligible patients from their routine patient base or new patient population. Digital X-ray facilities at the practice will be preferred but are not essential.

#### Identifying and recruiting participants

GDPs will identify patients presenting at their clinic/practice with symptoms indicative of irreversible pulpitis who meet the inclusion criteria. Patients with these symptoms who contact the practice by telephone will be informed the trial is taking place by the practice receptionist. An appointment will be arranged for potentially eligible patients (as per current clinical practice) for their treatment, and those who express interest in the feasibility study will be given a participant information leaflet (PIL) (Additional file 1). In the event that a patient presents at an appointment requiring immediate treatment, sufficient time to make an informed decision regarding willingness to participate will be given. At the treatment visit, the patient will be given the opportunity to clarify any questions prior to informed consent being provided on the study consent form (Additional file 2).

#### Allocation

Treatment options for irreversible pulpitis will be discussed with the patient and each recruited participant will be offered the FP treatment in the first instance. Should the dentist feel another treatment is more appropriate or a recruited participant changes their mind and does not want a FP, the appropriate alternative treatment should be carried out as per normal practice.

After the initial intervention, participants will receive any treatment deemed clinically appropriate by their dentist as per normal practice.

#### Inclusion criteria

One of the inclusion criteria is adults (16 years and older) with symptoms indicative of irreversible pulpitis in a pre/molar tooth with deep caries and/or a deep restoration as defined by ESE as spontaneous, radiating pain that lingers after removal of stimulus [11].

#### Exclusion criteria

The following are the exclusion criteria:

- Tooth with immature roots, clinical or radiographic signs of a necrotic pulp or a poor prognosis (e.g. internal or external resorption)
- Presence of a sinus, tenderness to percussion, buccal tenderness, pathological mobility or evidence of pathology on a periapical radiograph
- Insufficient tooth tissue for a restoration
- All treatment delivered under a private contract
- Unable to give informed consent

### Intervention

The following are the full pulpotomy procedure:

- Pre-operative peri-apical radiograph.
- Access cavity preparation.
- Where caries removal is necessary, this should be complete and carried out in a systematic way removing it completely at the periphery of the cavity then progressively over the pulp, for a controlled reduction in the bacterial load preventing further bacterial contamination of the pulp.
- Rubber dam should be applied prior to accessing the pulp chamber.
- Once the pulp has been reached, a new sterile bur or sharp sterile excavator should be used with a water coolant to remove the pulp to the level of the radicular/root canal orifices.
- The dentist should confirm that all root canals are vital.
- Haemostasis and disinfection should be achieved by placing cotton pellets soaked with 5% sodium hypochlorite over the pulp stump for up to 5 min.
- If haemostasis cannot be achieved after 5 min, the tooth should undergo pulpectomy and RCTx as per the clinician’s normal practice.
- Once haemostasis has been achieved for all root canals, a hydraulic calcium silicate cement should be placed directly onto the pulp tissue.
- The tooth is definitively sealed immediately to prevent micro-leakage. Immediate post-operative radiograph is taken for FP after the placement of definitive restoration. This radiograph will be used to confirm fidelity to the FP procedure to confirm that there is adequate coverage and thickness of hydraulic calcium silicate cement in the floor of the pulp chamber and pulp stumps and absence of porosity and the tooth is definitively sealed.

Clinical fidelity to the protocol will be assessed by the study’s clinical team. The clinical team will use the criteria below to evaluate the post-operative radiographs uploaded to the trial website by the participating dentists. Ultimately, the decision as to whether fidelity with the protocol has been maintained is a clinical judgement, made by the clinical team.

The following are the clinical fidelity criteria:

- Access cavity preparation (complete removal of the pulp chamber roof).
- Adaption of the calcium silicate cement (Biodentine). The cement should cover the floor or pulp stumps and have an adequate thickness of 2mm, and no porosities should be present.
- Adequate final restoration (no excess Biodentine on the walls preventing peripheral seal).

### Training in the delivery of intervention

Training will be provided over two sessions. One session will be a remote training session in which all dentists recruited to the feasibility study will receive training in the background and evidence for FP, diagnosis of irreversible pulpitis and study inclusion and exclusion criteria, along with theoretical training in the FP technique. The next session will be a face-to-face training session at each clinical centre. Study dentists will attend the face-to-face training session at their nearest clinical centre. We will confirm and identify the training needs of GDPs to inform the development of the training package for the full trial. PPI members will be invited to the remote training event to contribute to discussions of training and recruitment.

**Fig. 1:**
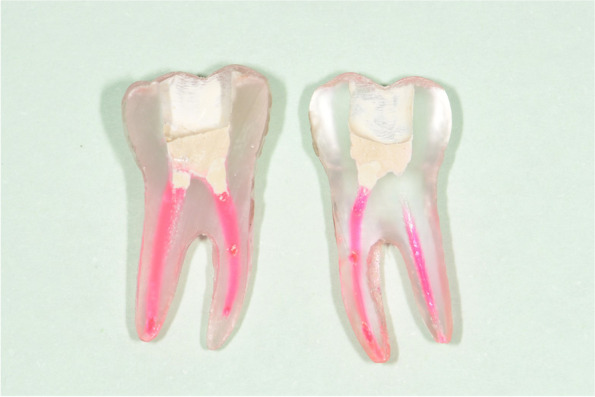
Example of the sectioned Endo Reality 3D tooth showing FP

As FP will be a new technology to most participants, instruction will demonstrate the procedure and establish the standard level of care. Instruction in access cavity preparation and choice of materials will include a hydraulic calcium silicate cement (e.g. Biodentine). Artificial 3D printed teeth (Fig. 1) are routinely used for RCTx training, but their use in the training in the FP technique and assessment of the success of training in the procedure is novel. We estimate 4 teeth for each dentist will be sufficient for them to feel confident and competent in the technique.

The participating GDPs’ success in training will be determined using the FP training criteria below to evaluate both the FP procedure carried out on the 3D teeth and the post-operative radiographs taken of the 3D teeth. Formative assessment for the dentists will include self-assessment using a success checklist measuring success against the same criteria. Ultimately, the decision as to whether a dentist has been successfully trained in the FP technique is a clinical judgement made by the study’s clinical team.

The following are the FP training criteria:

- Access cavity preparation (complete removal of the pulp chamber roof)
- Adaption of the calcium silicate cement (Biodentine) (covers the floor or pulp stumps, adequate thickness of 2mm, no porosity)
- Adequate final restoration (no excess calcium silicate cement (Biodentine) on the walls preventing peripheral seal)

### Primary outcome measures

Feasibility outcomes are set out in Table 1. These criteria will be assessed in the feasibility study in order to determine the progression to the main trial and a randomised controlled trial comparing the clinical-effectiveness and cost-benefit of FP compared to RCTx in pre/molar teeth of adults 16 years and older showing signs indicative of irreversible pulpitis.

**Table 1:**
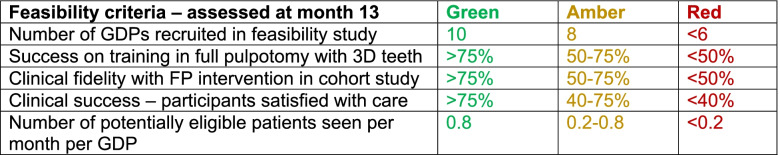
Feasibility criteria

Green—automatic progression. Amber—identify remediable factors and submit a recovery plan to the funder with new targets for the following 6 months. Red—stop the trial unless there is a strong case that unanticipated remediable factors have been identified and can be addressed

### Data collection and processing

An anonymised screening log has been created for the purposes of the PIP feasibility study data collection assessing the number of potentially eligible patients seen per month by each GDP.

Screening log data will either be entered into the database by the designated team members working in each site or sent to the Trial Office for entry into the database depending on practice circumstances. The staff in the Trial Office will work closely with the dental team members to ensure that the data are as complete and accurate as possible. Extensive range and consistency checks by the Trial Office will further enhance the quality of the data.

Participants who lose the capacity to consent during the study will be withdrawn. Identifiable data already collected with consent would be retained and used in the study. No further data will be collected or any other research procedures carried out on or in relation to the participant.

### Baseline characteristics

Age and gender will be recorded on the participant details form. Pre-operative periapical radiographs (if clinically needed) will be collected as part of routine care, but copies will be provided to the study team to confirm the exclusion of signs of pulpal and apical pathology.

### Trial process outcomes

The number of GDPs recruited will be assessed as the total number of GDPs that accept to take part in the study.

Success on training in FP with 3D teeth will be assessed according to the following criteria:

- Access cavity preparation (complete removal of the pulp chamber roof)
- Adaption of the calcium silicate cement (Biodentine) (covers the floor or pulp stumps, adequate thickness of 2mm, no porosity)
- Adequate final restoration (no excess calcium silicate cement (Biodentine) on the walls preventing peripheral seal)

The number of potentially eligible patients seen per month per GDP will be assessed as the total number of eligible patients recorded in the screening log by the GDP divided by the total number of GDPs taking part in the study.

### Clinical outcomes

Clinical fidelity with FP intervention in the feasibility study will be assessed by the study’s clinical team. The clinical team will use the criteria below to evaluate pre-operative and post-operative radiographs uploaded to the trial website by the participating dentists. Digital radiographs will be forwarded via the secure trial management system and digital images of wet films made. Ultimately, the decision as to whether fidelity with the protocol has been maintained is a clinical judgement, made by the clinical team. Each patient, from a given dentist, will be classified as complying with the clinical fidelity criteria or not. We will then calculate the percentage of patients, for each dentist, that comply with clinical fidelity. All dentists must reach the same clinical criteria set out in training to assess the feasibility of clinical fidelity with FP.

### Participant reported outcomes

Clinical success—participants satisfied with care—will be determined by patient-reported satisfaction with treatment using a question adapted from the patient-reported experience measures (PREMs) outlined in the NHS England Guide of Commissioning Dental Specialties [27]. The question is based on a scale ranging from not satisfied to completely satisfied. The intervention will be considered successful if the patient is somewhat to completely satisfied with their treatment. Participants will be contacted by their preferred method (phone, text or email) by the study office from day 7 to answer 2 questions on their satisfaction with care, experience and symptoms. Question 1: How satisfied are you with the treatment you received? Question 2: Did you have any problems in the hours after the treatment?

### Qualitative interviews

Qualitative interviews will be conducted with dentists and patient participants who have taken part in the feasibility study to explore the appropriateness of the training, the feasibility of delivering the interventions and the recruitment of participants to the trial. These will also contribute to the design of the trial recruitment strategies.

For patient participants, the qualitative interview will be part of the feasibility study, and all patient participants will have the option to consent to the interview as part of consenting to the feasibility study. If a patient participant gives their consent, a suitable date and time for a remote qualitative interview will be arranged after their follow-up questionnaire has been completed. The interview will be conducted by an experienced researcher and audio recorded.

All dentists will be invited to take part in an interview. Dentists will be contacted directly by the qualitative research team, informed about the qualitative study and invited to take part in a remote interview at a convenient time. Dentists will be contacted and interviewed at one of three time points: shortly after training, after recruiting 1–2 participants or after recruiting 3–4 participants in order to capture a range of experiences. Dentists will be asked to consent to take part in an interview.

The interviews will be guided by topic guides developed from the literature and other dental trials. The topic guide for dentists will be guided by the Theoretical Domains Framework [28] and focus on training, delivering the intervention, acceptability of the intervention and recruitment. The topic guide for patient participants will be informed by the Theoretical Framework of Acceptability [29], focusing on the experiences of recruitment and intervention, and acceptability of the intervention. Interviews will be audio-recorded and transcribed verbatim. The data will be analysed using framework analysis [30]. The framework analysis will involve the following stages: identifying initial themes, labelling the data, sorting the data by theme and synthesising the data. The interviews will be conducted by an experienced research associate who will also lead the data analysis. As the analysis progresses, regular meetings will be held with the research team to discuss the emergent themes and consider the implications of the results for the main trial.

### Process data

The details of treatment provided including adherence to the protocol and pulpotomy treatment or alternative treatment provided, duration of treatment, number of visits to deliver treatment, equipment used and patient charges will be recorded on the case report form (CRF).

Participants remain in the trial unless they choose to withdraw consent or if they are unable to continue for a clinical reason. All changes in status, with the exception of complete withdrawal of consent, means the participant will still be followed up for all trial outcomes wherever possible. All data collected through the screening log up to the point of complete withdrawal may be retained and used in the assessment of the feasibility study outcomes.

### Scheduling of events

The scheduling of events is presented in Table 2.

**Table 2:** Scheduling of events

	General dental practice approvals and training	Clinical intervention training	Screening	Baseline (initial treatment visit)	7-day follow-up	Qualitative interviews	Others
Number of general dental practitioners recruited	■	■					
Success on training in full pulpotomy		✓					
Assessment for eligibility			¥	o			
Informed consent				□			
Socio-demographic characteristics and eligibility for free treatment				o			
Clinical fidelity of the full pulpotomy intervention				o			●
Clinical success—participants satisfied with care					∞	⊕	
Dental pain and need for dental pain relief					∞		
Number of potentially eligible patients seen per month per GDP			∇				

“■” indicates general dental practitioner (GDP) signed off as greenlighted—completion of the Site Initiation Questionnaire, study approvals and training (Good Clinical Practice and clinical training)

“✓” indicates assessment by the study clinical team

“¥” indicates GDP completing the eligibility form

“o” indicates GDP completing case report form (CRF)

“□” indicates patient completing the consent form and countersigned by GDP

“●” indicates clinical post-operative radiographs

“∞” indicates phone call from the study team

“⊕” indicates qualitative interviews

“∇” indicates screening log

### Statistical analysis

#### Sample size calculation

Since this is a feasibility study and its aim is not to estimate a treatment effect, a sample size calculation was not performed. We aim to recruit 10 dentists and 40 patients because it was considered a large enough sample to inform training needs and recruitment to the main trial.

Demographic baseline characteristics, safety data and feasibility outcomes will be summarised overall and by centre if applicable and using appropriate descriptive statistics. The flow of participants will be presented as a diagram following adapted recommendations from the CONSORT extension for feasibility and pilot trials (https://www.bmj.com/content/bmj/355/bmj.i5239.full.pdf).

There are no planned interim analyses.

#### Patient and Public Involvement (PPI)

The feasibility study design was developed with input from a PPI partner on the project management group and the plain language summary and patient-facing materials developed with input from members of the Health Services Research Unit (HSRU) public involvement partnership. For example, for patient-facing documents such as the participant information leaflet and sheet and consent form, it was important to get the wording right around what the study is. Language such as ‘irreversible pulpitis’ was found to be detrimental, whereas ‘severe toothache which your dentist will assess’ was easier to understand. We have very good and thorough PPI representatives who also ensure consistent message delivery, alongside the study team. PPI partners will contribute patient and public perspectives at the dental training sessions.

A patient advisory group (facilitated by the PPI lead) will meet during the feasibility phase to give patient perspectives which can be incorporated in the main trial development, and advise on content and routes for engagement with and dissemination to patients and the public. Additional PPI input will be provided by two PPI partners on the independent steering committee. The PPI lead and will support researchers and PPI partners throughout the trial.

### Ethical conduct of the trial

The trial will run under the auspices of the trial office in Dundee Dental School and CHaRT in the University of Aberdeen. CHaRT is a fully registered Clinical Trials Unit with extensive expertise in running multicentre RCTs. Both institutions are committed to the highest standards of research governance and conform to all relevant governance guidelines and codes of practice as detailed in the Research Governance Framework and ICH guidelines for Good Clinical Practice (GCP).

### Data protection and archiving

Patients will be reassured that all data which are collected during the course of the research will be kept strictly confidential. All personal data will be pseudonymised and processed in accordance with the General Data Protection Regulation Act 2018. The relevant research documentation will be archived at the University of Dundee for at least 10 years after completion of the trial as required by the applicable regulatory requirement(s).

### Governance arrangements

Research governance applies to everyone working in the Dental Health Services & Research Unit and CHaRT. As such, all research will be conducted within the appropriate legislative and regulatory environment and in accordance with GCP. All staff involved in the trial at the two centres will have undertaken appropriate GCP training (to a level of knowledge that reflects their exposure to the principles). The three main groupings that contribute to the governance arrangements for this study are the Trial Management Committee, an independent Trial Steering Committee (TSC) and an independent Data Monitoring Committee (DMC). The TSC and DMC will meet during the feasibility study to agree with the terms of reference and other procedures. The DMEC will report any recommendations to the Chair of the Steering Committee. The University of Dundee will act as a sponsor.

### Arrangements for the day-to-day management of the trial

The TCOD based in the Dundee Dental School at the University of Dundee will provide day-to-day support for the clinical centres and sites. CHaRT, Health Services Research Unit, Aberdeen University, will provide the database applications and IT programming for the TCOD and provide experienced trial management guidance. The principal investigators (GDPs) will be responsible for recruiting participants and performing full pulpotomy treatment.

The study will be supervised by a Project Management Group (PMG). The co-chairs of this group will be the co-chief investigators and will consist of grant holders and representatives from the TCOD and CHaRT. The PMG will meet at least monthly; however, meetings may be more frequent.

### Safety concerns

Within the PIP feasibility study, only adverse events (AEs) and serious adverse events (SAEs), as determined by the site PI, that have a reasonable causal relationship to the FP treatment in the study tooth will be recorded. These will be reported to the TM who will report/escalate as necessary.

### Adverse events

Whilst a FP is a novel treatment in NHS primary care clinical practice, in terms of clinical procedure, it is more conservative than the established RCTx and could be considered as the same technique that is used in the initial stage of a RCTx. We do not anticipate any safety concerns with this treatment. The dentists taking part in the feasibility study will be fully trained in the FP technique, and patients will receive the usual standard of care treatment from their dentist during and following the intervention as normal.

The following adverse events are not common but potentially expected:

- Further failure of tooth vitality with associated signs or symptoms (e.g. pain, infection, swelling, periodontitis)
- Failure due to peri-radicular pathology with associated signs or symptoms (e.g. pain, infection, swelling, periodontitis)
- Dental infection associated with the feasibility study tooth
- Further treatment required (under local anaesthetic and/or general anaesthetic)
- Perforation
- Hypochlorite leakage into the oral cavity
- Hypochlorite injury

### Recording and reporting of adverse events

From the time a participant consents to join the study until the end of their follow-up, SAEs will be recorded on an SAE form. Events that are serious but are not related to full pulpotomy in the trial tooth will not be recorded as SAEs. The local investigator (PI) should make an assessment of seriousness as per the definitions of adverse events and serious adverse events. SAEs will be recorded and reported to the sponsor within 24 h of becoming aware of the event and further follow-up information provided as soon as available. Site PI will determine whether it is an AE. Report to TM who will report/escalate as necessary.

### Publication

The results of the study will be reported first to study collaborators. The main report will be drafted by the PMG and circulated to all clinical coordinators for comment before a final version is considered for publication by the steering committee.

### Dissemination and outputs

On completion of the feasibility study, the feasibility study data will be analysed and tabulated, and a clinical trial report will be prepared. The findings of the feasibility study will be published in a peer-reviewed journal.

On completion of the feasibility study, if it has been determined that progression to the main trial is possible, the data from the feasibility study will be analysed and tabulated, and a clinical trial report will be prepared in conjunction with the clinical report of the main trial.

This feasibility study investigates a treatment option identified as being potentially able to generate a significant cost saving for the NHS. We have found it to be of high interest to practitioners and patients. We will produce new knowledge which will be valuable for these and other key stakeholder groups both in the UK and internationally. We will use varied communication strategies to ensure that all stakeholder groups are updated throughout the feasibility study and aware of the feasibility study outcome.

#### NHS

The results of the feasibility study will be communicated directly to all participating dental practices. Members of the team may speak about the feasibility study at national conferences for GDPs such as the British Dental Association conference, meetings and conferences of the Faculty of General Dental Practitioners and local practitioner meetings. Our experience of conducting the feasibility study will be used alongside our successful approach of including participating practitioners to speak at meetings, giving them an opportunity to raise awareness of the rewards of research participation as well as increasing visibility of the main trial. We will produce clinical summary papers for clinician-targeted journals.

#### End of trial dissemination events

Final dissemination events will be organised to report on the decision to proceed to full trial after the feasibility trial end. This will include key stakeholders (e.g. patients/national patient advocates, clinicians, NHS England commissioners, GDP providers participating practices/participants) to deliver the impact across a wide audience.

### Milestones for the PIP Trial

**Fig. 2:**
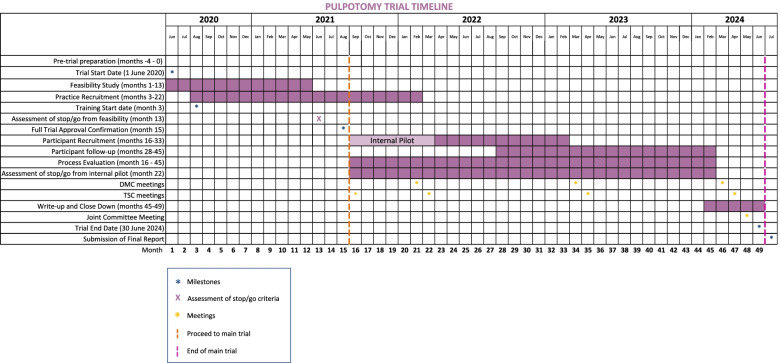
Gantt chart outlining the project timeline and milestones

Project timeline and milestones are outlined in Fig. 2

Dental practice recruitment began on 9 October 2020. Follow-up assessments will take place 7 days after delivery of the FP.

## Discussion

The PIP Trial is an NIHR HTA-funded trial being undertaken across the UK and will begin to address the lack of high-quality evidence to aid dental practitioners, patients and policymakers in their decision-making. The PIP feasibility study will inform the main study, which will be a pragmatic, multi-centre, randomised, open trial with blinded outcome evaluation. PIP aims to eradicate the uncertainty that exists among dental practitioners when treating irreversible pulpitis by testing the interventions in the environment that they will most often be delivered in, dental primary care.

In order to ensure the results of this trial are widely applicable, the geographical areas that are included in the PIP Trial have been selected to yield a cross-section of practices, operating in a range of different environments and circumstances (e.g. high-, middle- or low-income communities), rural and urban, method of remuneration for GDPs (capitation and fee for item of service or a banded payment system based on Units of Dental Activity (UDA)).

The study team is multidisciplinary and broad-based and will be led by the teams at the Dental Health Services Research Unit, Dundee, and the Centre for Healthcare Randomised Trials in Aberdeen. This will ensure that whilst the trial design and conduct is of the highest standard, it remains practical and pragmatic at all times.

## Supplementary Information

**Additional file 1.** Participant information leaflet (PIL).

**Additional file 2.** Participant consent form.

## Data Availability

The final trial data datasets will be available from chief investigator Jan Clarkson on reasonable request.

## Abbreviations

AE: Adverse event
CCR: Complete caries removal
CHaRT: Centre for Healthcare Randomised Trials
CI: Chief investigator
CONSORT: Consolidated Standards of Reporting Trials
CRF: Case report form
CTU: Clinical trial unit
DCE: Discrete choice experiment
DMC: Data Monitoring Committee
DMS: Data Management System
EQ-5D-5L: EuroQOL Five Dimensions Questionnaire
GCP: Good Clinical Practice
GDP: General dental practitioner
HRQoL: Health-related quality of life
HSRU: Health Services Research Unit
HTA: Health technology assessment
ICF: Informed consent form
ISD: Information Statistics Division
ISF: Investigator site file
ISRCTN: International Standard Randomised Controlled Trial Number
IVR: Interactive voice response (randomisation)
OMG: Operations Management Group
MRC: Medical Research Council
NCT: National Clinical Trial
NHS: National Health Service
NIHR: National Institute Health Research
PI: Principal investigator
PIL: Patient information leaflet
PMG: Project Management Group
PPI: Patient and Public Involvement
PQ: Patient questionnaire
QALY: Quality-adjusted life year
RCT: Randomised controlled trial
R&D: Research and Development
REC: Research Ethics Committee
SAE: Serious adverse event
SAP: Statistical analysis plan
SCR: Selective caries removal
SD: Standard deviation
SOP: Standard operating procedures
TCOD: Trial Coordinating Office Dundee
TMF: Trial Master File
TSC: Trial Steering Committee
UK: United Kingdom
UKCRC: United Kingdom Clinical Research Collaboration
UoA: University of Aberdeen
