# Correction to: Pulpotomy for the Management of Irreversible Pulpitis in Mature Teeth (PIP): a feasibility study

**DOI:** 10.1186/s40814-022-01045-9

**Published:** 2022-04-11

**Authors:** Jan E. Clarkson, Craig R. Ramsay, Francesco Mannocci, Fadi Jarad, Sondos Albadri, David Ricketts, Carol Tait, Avijit Banerjee, Chris Deery, Dwayne Boyers, Zoe Marshman, Beatriz Goulao, Alice R. Hamilton, Katie Banister, Rosanne Bell, Lori Brown, David I. Conway, Pina Donaldson, Anne Duncan, Katharine Dunn, Patrick Fee, Mark Forrest, Anne-Marie Glenny, Jill Gouick, Ekta Gupta, Elisabet Jacobsen, Jennifer Kettle, Graeme MacLennan, Lorna Macpherson, Tina McGuff, Fiona Mitchell, Marjon van der Pol, Rebecca Moazzez, Douglas Roberston, Gabriella Wojewodka, Linda Young, Thomas Lamont

**Affiliations:** 1 Dental Health Services Research Unit, Dundee Dental School, The University of Dundee, 9th Floor, Park Place, Dundee, DD1 4HN UK; 2 NHS Education for Scotland, Edinburgh, UK; 3 Health Services Research Unit, University of Aberdeen, Aberdeen, UK; 4 Centre for clinical and translational sciences, King’s College London, London, UK; 5 School of Dentistry, University of Liverpool, Liverpool, UK; 6 Dundee Dental School, The University of Dundee, Dundee, UK; 7 Faculty of Dentistry, Oral & Craniofacial Services, Kings College London, London, UK; 8 School of Clinical Dentistry, University of Sheffield, Sheffield, UK; 9 Health Economics Research Unit, University of Aberdeen, Aberdeen, UK; 10 Patient and Public Involvement Health Services Research Unit, University of Aberdeen, Aberdeen, UK; 11 School of Medicine, Dentistry and Nursing, University of Glasgow, Glasgow, UK; 12 Edinburgh Dental Institute, NHS Lothian, Edinburgh, UK; 13 Division of Dentistry, University of Manchester, Manchester, UK; 14 Oral Clinical Research Unit, King’s College London, London, UK

**Correction to: Pilot Feasibility Stud 8, 77 (2022)**

**https://doi.org/10.1186/s40814-022-01029-9**

Following publication of the original article [1], the authors reported an error in the author group. Author Alice Hamilton is listed twice in the author group, she is listed as the 1st author and 14th author. Author “Jan Clarkson” should be to be listed as the first author and the current first author “Alice Hamilton” should be removed.

The original article [1] has been updated.
