# Supplementary material for: Pulpotomy for the Management of Irreversible Pulpitis in Mature Teeth (PIP): a feasibility study
Source: Pilot Feasibility Stud. 2022 Apr 2;8:77. doi: 10.1186/s40814-022-01029-9 (PMC8976106; doi:10.1186/s40814-022-01029-9)
Supplement: Supplementary file 1 — Additional file 1. Participant information leaflet (PIL). [file 40814_2022_1029_MOESM1_ESM.pdf]

Study of a new treatment for toothache called Pulpotomy

IRAS number 289464

Participant Information Leaflet

INVITATION TO TAKE PART

We would like to invite you to take part in a dental research study.

The following information explains more about the study.

Part A gives an overview of what taking part would mean for you

Part B answers some of the questions you might have

Part C gives further information about the study and how we use the data you give us

Part D gives further information about how health researchers use your data

Please take time to read the information.

Feel free to talk to your friends and family about taking part.

Speak to your dentist if there is anything that is not clear or if you would like more information

FUNDED BY
NIHR | National Institute for Health Research

Part A: Patient Journey

Are you interested in taking part in a dental research study?

You could take part at your treatment appointment!

If you choose to take part

You will complete and sign a **Consent Form**

Treatment:
You will have the new Pulpotomy Treatment

7 days after treatment:
the research team will be in touch with **2 short questions** about your satisfaction with the treatment.

1-4 weeks after treatment:
the research team will have a **longer conversation** with you about your treatment experiences (approx. 20-40 minutes)
optional

To thank you for your support, you will receive a £15 gift voucher at the beginning and £15 at the end of the study.

If you choose not to take part

Treatment:
You will have Root Canal Treatment or have your tooth removed

You will have regular check-ups as usual with your dentist.

Interested in knowing more?

Please read Part B, C & D of this leaflet and talk to the Dental Practice Team

Part B: Questions you might have

What is the purpose of the research study?

Severe toothache and pain can be caused by tooth decay. Tooth decay is very common. Where the tooth decay has spread deep into the nerve, this is usually treated by taking out the tooth or by Root Canal Treatment. Root Canal Treatment removes all of the nerve from the tooth.

An alternative treatment for this kind of toothache is called a Pulpotomy. A Pulpotomy removes only the damaged part of the nerve, which can be a quicker and less invasive treatment. Pulpotomy is already used on children's teeth and on adult teeth when they are being treated in dental hospitals. However it is not yet routinely used on adult teeth in NHS dental practices.

We are planning a large-scale study to compare Pulpotomy treatment with Root Canal Treatment in NHS dental practices. However, before we do a large study we want to find out more about the patient and dentist experiences of the new treatment (Pulpotomy). We are doing this by running this smaller study called a feasibility study. It will help us to take patients' and dentists' experiences into account when planning the larger study.

It will help us find out more about:

a) how many patients might need this kind of treatment
b) how many patients might be interested in the Pulpotomy treatment
c) the practicalities of running this kind of study in NHS dental practices
d) how best to train dentists to deliver the Pulpotomy treatment

We need to ensure that research meets patient needs and are asking your help to do this. By collecting information, this study will help us to decide whether a larger scale study to compare treatments is feasible and the best way to run the large scale study.

Your dentist has signed up to take part in this study as they are interested in knowing if a Pulpotomy can be delivered in primary care dental practices like yours. They have been trained to deliver this treatment by specialist dentists.

Why are you asking me?

Your toothache needs treatment which would normally be done by Root Canal Treatment, or by taking the tooth out. Your dentist believes that the Pulpotomy treatment would also be suitable for you and thinks you meet the study criteria to have the treament. We are asking a number of people around the country who have toothache like yours which needs treatment, to take part in the study.

What will happen if I say yes?

1. **Your dentist will ask you to sign a consent form.**
   This form is used to record that you understand the study and what will happen. You will be given a copy of the form to keep, as well as this information sheet.
2. **You will then have the <u>Pulpotomy</u> treatment on your tooth**.
   A pulpotomy removes only the damaged part of the nerve, which can be a quicker and less invasive treatment. Your dentist will work carefully to gain access to the nerve of your tooth in the same way as they would for a Root Canal Treatment. They will then only remove the damaged part of the nerve

and place a filling over the top. This means that some of the nerve of your tooth will remain alive. You will pay for your treatment as you normally would.

3. **We will contact you around 7 days after your treatment to ask you two questions about your treatment.**
4. **If you choose to take part in the patient interview, you will have a longer conversation with our research team**. You will be asked to talk about your experience of dental treatment in the study and your thoughts about the planned larger study. This will last approximately 20-40 minutes depending on how much you would like to say. The conversation will be arranged at a suitable time for you within a month of you having your treatment and can be done over the phone or by video call.

What are the possible advantages or disadvantages of taking part?

If you take part you will receive Pulpotomy treatment for your toothache. You might not gain any additional benefit from taking part. The Pulpotomy treatment may be quicker and less invasive than other treatments such as Root Canal Treatment and is an alternative to having an extraction.
As with all treatments, there is a small risk that the treatment may not work and you may require further treatment. This is also the case if you were to opt for a Root Canal Treatment"

The full pulpotomy treatment includes having X-rays taken. This is part of your routine care, and would be the same if you were to opt for a Root Canal Treatment. Dental radiographs need very low doses of radiation, and are equivalent to less than a day of background radiation.

You will be directly helping the treatment of future patients with toothache like yours. The results of this study and your feedback will help us to design a larger study with more dentists and patients in NHS dental practices across the UK.

You will receive £30 in gift vouchers; £15 at the start and £15 at the end of your time in the study.

Who will know I am in this study?

You will be given a special identification number and any information we collect for the study will be stored using this number, rather than using any personal details. This means that only the people who are treating you, or who need to contact you, will know who you really are. In this study we will use information from you and your dental records. We will only use information that we need for the study. We will let very few people know your name or contact details, and only if they really need it for this study.

Everyone involved in this study will keep your data safe and secure and will follow all privacy rules. At the end of the study we will save some of the data for future research. We will make sure no-one can work out who you are from the reports we write. The information pack part C and D tells you more about this.

Do I have to say yes?

No you don't. If you decide to say no, nobody will mind, and you and your dentist can proceed with treating your toothache as they normally would.

What happens if I change my mind?

You will still be treated by your dentist as normal. If you do change your mind about taking part, please tell your dentist or the study team and the study team won't contact you again.

Who is running the study?

This study is being run by a team of experienced dentists and researchers, led by Professor Jan Clarkson at the Dundee Dental School. The study is sponsored by the University of Dundee and has been funded by the National Institute for Health Research Health Technology Assessment (www.nihr.ac.uk).

How can I find out more?

If you have any questions or worries about the information in this leaflet or anything else related to the study please speak to your dentist. Further details of the study are available online at https://w3.abdn.ac.uk/hsru/PIP/Public/Public/index.cshtml

PIP Feasibility study Office, Level 9, School of Dentistry, University of Dundee, Dundee Dental Hospital, Park Place, DUNDEE Scotland DD1 4HN Tel: 01382 831727, Email: PIP-Study@dundee.ac.uk

Part C: Further Information

Who is eligible to take part?

Adults (16 and over) who receive some or all their care through the NHS and have been diagnosed with toothache caused by decay in their tooth that requires treatment may be eligible. Eligibility will be confirmed by your dentist.

Who has reviewed this study?

All research in the NHS is looked at by an independent group of people, called a Research Ethics Committee (REC), to protect your interests. This study has been reviewed and given favourable opinion by the West of Scotland Research Ethics Service

How have patients and the public been involved in this study?

Members of the public are part of the research team throughout the study and help make sure the research is important to patients. They have also helped with drafting this participant information sheet.

What will happen to the results of this study?

The results of the study will be used to decide whether we can progress to a full study recruiting more practices and patients. We may publish the results of this study in scientific journals and present the information at appropriate meetings. You will not be identified in any publication of results of the study. We will let you know the results of the study when it is finished unless you tell us that you do not wish to know.

What happens when the research study stops?

Your dentist will continue to provide your dental care after the study has ended. Your future dental care will be at the discretion of your own dentist. You will be able to access the results of the study once they are published and we will provide you with details about how to access this information. If for any reason the study ends earlier than expected, we will let you know. Your continuing dental care will carry on as normal.

What if relevant new information becomes available?

Sometimes during the course of a research project, new information becomes available about the treatment that is being studied. If this happens, the study office staff will contact you to let you know about the choices available to you. However, we are not aware that any new, relevant information is likely to become available before the end of this study.

What will happen if I don't want to carry on with the study?

You are free to decide at any time to withdraw from the study. This decision will not affect the standard of care you are receiving now or in the future. If you make this decision, you should continue attending appointments with your dentist as part of your standard care.

If you decide to withdraw from this study, we will keep and continue to use your previously collected data. We will, however not collect any further data about you. This information will remain confidential and will use the minimum personally-identifiable information possible.

Part C: Further Information

Will my taking part in the study be kept confidential?

Yes. We will follow ethical and legal practice and all information which is collected about you for the purpose of this research study will be handled in strict confidence and securely stored by the Universities of Aberdeen, Dundee and Sheffield. Only specified members of the research team will have access to your information.

Personal information and routine national data

We will keep all information about you safe and secure. The University of Dundee will collect information about you for this study from you and your dentist. We will need to use information from you and from your dental records for this research project.
This information will include:

- your name
- NHS number/ CHI number
- contact details (address, email address, telephone numbers)
- date of birth

We will only use this information to conduct this study. People will use this information to do the research or to check your records to make sure that the research is being done properly. People who do not need to know who you are will not be able to see your name or contact details. Your data will have a code number instead.

The interview will be carried out by a researcher from the University of Sheffield. Your interview will be held via telephone or video call. It will be audio recorded and transcribed. Once the transcript has been checked, your audio recording will be deleted. Your transcript will be stored by the University of Sheffield. It will not include your name, and will be stored under a code number. Quotes from your transcript may be used in written reports and presentations. These will not include your name.

We will review national health registers to find out about relevant treatments you have received and the progress of your tooth after the study has ended. To carry this out we need to send some information about you to national health registers (e.g. NHS Business Services Authority, HSC Business Services Organisation (BSO), NHS National Services Scotland (NSS), Information Services Division (ISD), Office of National Statistics (ONS), NHS Digital). This information would then be matched to their records about dental treatments, and then returned to the statisticians at the University of Aberdeen where it will be anonymised. All information would be sent and stored securely throughout the process.

Once we have finished the study, we will keep some of the data so we can check the results. We will write our reports in a way that no-one can work out that you took part in the study.

What are your choices about how your information is used?

- You can stop being part of the study at any time, without giving a reason, but we will keep information about you that we already have.
- If you choose to stop taking part in the study, we would like to continue collecting information about your health from central NHS records and your dentist. If you do not want this to happen, tell us and we will stop.
- We need to manage your records in specific ways for the research to be reliable. This means that we won't be able to let you see or change the data we hold about you.
- If you agree to take part in this study, you will have the option to take part in future research using your data saved from this study.

Where can you find out more about how your information is used?

You can find out more about how we use your information

- at https://www.hra.nhs.uk/information-about-patients/
- our leaflet available from the research sponsor https://www.dundee.ac.uk/information-governance/dataprotection/
- by asking one of the research team at your dentist or the PIP Study Office
- by sending an email to PIP-study@dundee.ac.uk

What if there is a problem?

If you are concerned about your participation in the study you have the right to discuss your concern with a researcher involved in carrying out the study or a dentist involved in your care. If you have a complaint about your participation in the study first of all you should talk to a researcher involved in the study or your dentist. You can also make a formal complaint to the Complaints Officer for your local NHS Health Board or NHS Clinical Commissioning Group.

The University of Dundee holds and maintains policies of insurance for legal liabilities arising from the study.

If you are harmed due to someone's negligence, then as a patient of the NHS, you may have grounds for legal action. You may have to pay for your legal costs yourself.

Part D: GDPR information from Health Research Authority

The following information explains how health researchers use information from patients. If you are asked to take part in research, you can ask what will happen in the study.

What is patient data?

When you go to your GP or hospital, the doctors and others looking after you will record information about your health. This will include your health problems, and the tests and treatment you have had. They might want to know about family history, if you smoke or what work you do. All this information that is recorded about you is called patient data or patient information.

When information about your health care joins together with information that can show who you are (like your name or NHS number) it is called identifiable patient information. It's important to all of us that this identifiable patient information is kept confidential to the patient and the people who need to know relevant bits of that information to look after the patient. There are special rules to keep confidential patient information safe and secure.

What sort of patient data does health and care research use?

There are lots of different types of health and care research.
If you take part in a clinical trial, researchers will be testing a medicine or other treatment. Or you may take part in a research study where you have some health tests or answer some questions. When you have agreed to take part in the study, the research team may look at your medical history and ask you questions to see if you are suitable for the study. During the study you may have blood tests or other health checks, and you may complete questionnaires. The research team will record this data in special forms and combine it with the information from everyone else in the study. This recorded information is research data.

In other types of research, you won't need to do anything different, but the research team will be looking at some of your health records. This sort of research may use some data from your GP, hospital or central NHS records. Some research will combine these records with information from other places, like schools or social care. The information that the researcher collects from the health records is research data.

Why does health and care research use information from patients?

In clinical trials, the researchers are collecting data that will tell them whether one treatment is better or worse than another. The information they collect will show how safe a treatment is, or whether it is making a difference to your health. Different people can respond differently to a treatment. By collecting information from lots of people, researchers can use statistics to work out what effect a treatment is having.

Other types of research will collect data from lots of health records to look for patterns. It might be looking to see if any problems happen more in patients taking a medicine. Or to see if people who have screening tests are more likely to stay healthier.

Some research will use blood tests or samples along with information about the patient's health. Researchers may be looking at changes in cells or chemicals due to a disease.

All research should only use the patient data that it really needs to do the research. You can ask what parts of your health records will be looked at.

How does research use patient data?

If you take part in some types of research, like clinical trials, some of the research team will need to know your name and contact details so they can contact you about your research appointments, or to send you questionnaires. Researchers must always make sure that as few people as possible can see this sort of information that can show who you are.

In lots of research, most of the research team will not need to know your name. In these cases, someone will remove your name from the research data and replace it with a code number. This is called coded data, or the technical term is pseudonymised data. For example, your blood test might be labelled with your code number instead of your name. It can be matched up with the rest of the data relating to you by the code number.

In other research, only the doctor copying the data from your health records will know your name. They will replace your name with a code number. They will also make sure that any other information that could show who you are is removed. For example, instead of using your date of birth they will give the research team your age. When there is no information that could show who you are, this is called anonymous data.

Where will my data go?

Sometimes your own doctor or care team will be involved in doing a research study. Often, they will be part of a bigger research team. This may involve other hospitals, or universities or companies developing new treatments. Sometimes parts of the research team will be in other countries. You can ask about where your data will go. You can also check whether the data they get will include information that could show who you are. Research teams in other countries must stick to the rules that the UK use.

All the computers storing patient data must meet special security arrangements.

If you want to find out more about how companies develop and sell new medicines, the Association of the British Pharmaceutical Industry has information on its website https://www.abpi.org.uk/.

What are my choices about my patient data?

- You can stop being part of a research study at any time, without giving a reason, but the research team will keep the research data about you that they already have. You can find out what would happen with your data before you agree to take part in a study.
- In some studies, once you have finished treatment the research team will continue to collect some information from your doctor or from central NHS records over a few months or years so the research team can track your health. If you do not want this to happen, you can say you want to stop any more information being collected.
- Researchers need to manage your records in specific ways for the research to be reliable. This means that they won't be able to let you see or change the data they hold about you. Research could go wrong if data is removed or changed.

What happens to my research data after the study?

Researchers must make sure they write the reports about the study in a way that no-one can work out that you took part in the study.

Once they have finished the study, the research team will keep the research data for several years, in case they need to check it. You can ask about who will keep it, whether it includes your name, and how long they will keep it.

Usually your hospital or GP where you are taking part in the study will keep a copy of the research data along with your name. The organisation running the research will usually only keep a coded copy of your research data, without your name included. This is kept so the results can be checked.

If you agree to take part in a research study, you may get the choice to give your research data from this study for future research. Sometimes this future research may use research data that has had your name and NHS number removed. Or it may use research data that could show who you are. You will be told what options there are. You will get details if your research data will be joined up with other information about you or your health, such as from your GP or social services.

Once your details like your name or NHS number have been removed, other researchers won't be able to contact you to ask you about future research.

Any information that could show who you are will be held safely with strict limits on who can access it.

You may also have the choice for the hospital or researchers to keep your contact details and some of your health information, so they can invite you to take part in future clinical trials or other studies. Your data will not be used to sell you anything. It will not be given to other organisations or companies except for research.

Will the use of my data meet GDPR rules?

GDPR stands for the General Data Protection Regulation. In the UK we follow the GDPR rules and have a law called the Data Protection Act. All research using patient data must follow UK laws and rules.

Universities, NHS organisations and companies may use patient data to do research to make health and care better.

When companies do research to develop new treatments, they need to be able to prove that they need to use patient data for the research, and that they need to do the research to develop new treatments. In legal terms this means that they have a 'legitimate interest' in using patient data.

Universities and the NHS are funded from taxes and they are expected to do research as part of their job. They still need to be able to prove that they need to use patient data for the research. In legal terms this means that they use patient data as part of 'a task in the public interest'. If they could do the research without using patient data they would not be allowed to get your data.

Researchers must show that their research takes account of the views of patients and ordinary members of the public. They must also show how they protect the privacy of the people who take part. An NHS research ethics committee checks this before the research starts.

What if I don't want my patient data used for research?

You will have a choice about taking part in a clinical trial testing a treatment. If you choose not to take part, that is fine.
In most cases you will also have a choice about your patient data being used for other types of research. There are two cases where this might not happen:

1. When the research is using anonymous information. Because it's anonymous, the research team don't know whose data it is and can't ask you.
2. When it would not be possible for the research team to ask everyone. This would usually be because of the number of people who would have to be contacted. Sometimes it will be because the research could be biased if some people chose not to agree. In this case a special NHS group will check that the reasons are valid. You can opt-out of your data being used for this sort of research. You can ask your GP about opting-out, or you can find out more by visiting https://www.hra.nhs.uk/information-about-patients/.

Who can I contact if I have a complaint?

If you want to complain about how researchers have handled your information, you should contact the research team. If you are not happy after that, you can contact the Data Protection Officer. The research team can give you details of the right Data Protection Officer.

If you are not happy with their response or believe they are processing your data in a way that is not right or lawful, you can complain to the Information Commissioner's Office (ICO) (https://www.ico.org.uk or 0303 123 1113).

Thank you for taking the time to read this information leaflet. We hope that it has been helpful in enabling you to decide if you would like to participate in this study. Please ask us if you have questions or would like more information about the study.
