# Supplementary material for: Pulpotomy for the Management of Irreversible Pulpitis in Mature Teeth (PIP): a feasibility study
Source: Pilot Feasibility Stud. 2022 Apr 2;8:77. doi: 10.1186/s40814-022-01029-9 (PMC8976106; doi:10.1186/s40814-022-01029-9)
Supplement: Supplementary file 2 — Additional file 2. Participant consent form. [file 40814_2022_1029_MOESM2_ESM.pdf]

Study No			

PIP Feasibility Study: Pulpotomy for Irreversible Pulpitis

PARTICIPANT CONSENT FORM

IRAS: 289464

Investigators: Prof Jan Clarkson, University of Dundee; Prof Craig Ramsay, University of Aberdeen
Sponsor: University of Dundee

If you **agree** to the following sentences, please ***initial*** next to each statement:

Please **INITIAL** each point

1. I have read the Participant Information Leaflet (parts A, B, C, D) [version 1, 26.01.2021] for the PIP Feasibility Study. I have had the opportunity to think about the information, ask questions and my questions have been answered. ☐
2. I understand that it is my choice to take part. I also understand that I can change my mind at any time in the future and without giving a reason and understand doing so will not affect my dental care or legal rights. ☐
3. I understand personal information that identifies me collected during the Feasibility Study, together with my personal contact details, will be confidentially and securely stored by the Universities of Dundee, Aberdeen and Sheffield. I agree that the research team can use my contact details to contact me by phone, post, email or text regarding the Feasibility Study. ☐
4. I agree that personal information (such as my name, date of birth, home postcode and NHS/CHI number) can be used to find out other information about my dental health that is collected by the NHS, including information from national dental payment databases (e.g. NHS Business Services Authority, NHS National Services Scotland (NSS), Public Health Scotland, Office of National Statistics (ONS), NHS Digital). ☐
5. I agree that relevant sections of my dental notes and dental information collected during the study may be looked at by people directly involved in the study (at the Universities of Dundee, Aberdeen and Sheffield or in the NHS) where it is relevant to my taking part in this research. ☐

I agree to take part in the PIP Study ☐

OPTIONAL (You do not have to give consent to this statement to take part in PIP)

6. I am willing to be contacted to take part in a short interview about my treatment, which will be audio recorded ☐
7. I am willing to be contacted in the future for long-term follow-up. ☐

PARTICIPANT SIGNATURE

Your Signature	Name in BLOCK CAPITALS	Date

For Dental Practice Staff Use Only
SIGNATURE OF PERSON TAKING CONSENT

Job Title	Name in BLOCK CAPITALS	Signature	Date

IT IS NOW ESSENTIAL THAT YOU RECORD THIS CONSENT FORM ON THE PIP DATABASE

PIP Feasibility Study Office Dundee, Level 9, School of Dentistry, University of Dundee, Dundee Dental Hospital, Park Place, DUNDEE, Scotland, DD1 4HN Tel: 01382 388551 or 01382 381727, Email: PIP-Study@dundee.ac.uk
COPIES: Original (top copy): For Study Office. **Yellow copy:** For practice site file. **Pink copy:** For patient participant.
